# Neural Transformation from Retinotopic to Background-Centric Coordinates in the Macaque Precuneus

**DOI:** 10.1523/JNEUROSCI.0892-24.2024

**Published:** 2024-10-15

**Authors:** Motoaki Uchimura, Hironori Kumano, Shigeru Kitazawa

**Affiliations:** ^1^Dynamic Brain Network Laboratory, Graduate School of Frontier Biosciences, Osaka University, 1-3 Yamadaoka, Suita, Osaka 565-0871, Japan; ^2^Department of Integrative Physiology, Graduate School of Medicine, University of Yamanashi, 1110 Shimokato, Chuo, Yamanashi 409-3898, Japan; ^3^Department of Brain Physiology, Graduate School of Medicine, Osaka University, 1-3 Yamadaoka, Suita, Osaka 565-0871, Japan; ^4^Center for Information and Neural Networks (CiNet), National Institute of Information and Communications Technology, 1-4 Yamadaoka, Suita, Osaka 565-0871, Japan

**Keywords:** allocentric coordinate, background coordinate, default-mode network, precuneus, retinotopic coordinate, visual stability

## Abstract

Visual information is initially represented in retinotopic coordinates and later in craniotopic coordinates. Psychophysical evidence suggests that visual information is further represented in more general coordinates related to the external world; however, the neural basis of nonegocentric coordinates remains elusive. This study investigates the automatic transformation from egocentric to nonegocentric coordinates in the macaque precuneus (two males, one female), identified by a functional imaging study as a key area for nonegocentric representation. We found that 6.2% of neurons in the precuneus have receptive fields (RFs) anchored to the background rather than to the retina or the head, while 16% had traditional retinotopic RFs. Notably, these two types were not exclusive: many background-centric neurons initially encode a stimulus' position in retinotopic coordinates (up to ∼90 ms from the stimulus onset) but later shift to background coordinates, peaking at ∼150 ms. Regarding retinotopic information, the stimulus dominated the initial period, whereas the background dominated the later period. In the absence of a background, there is a dramatic surge in retinotopic information about the stimulus during the later phase, clearly delineating two distinct periods of retinotopic encoding: one focusing on the figure to be attended and another on the background. These findings suggest that the initial retinotopic information of the stimulus is combined with the background retinotopic information in a subsequent stage, yielding a more stable representation of the stimulus relative to the background through time-division multiplexing.

## Significance Statement

According to psychological literature, the location of visual stimuli is automatically positioned against the background of a scene. This representation relative to the background, not being influenced by eye movements, should be important for stabilizing the visual world. A human functional imaging study suggested that the precuneus in the medial cerebral cortex is a strong candidate. This study recorded neural activity from the precuneus of monkeys and demonstrated the existence of background-centered cells with receptive fields fixed relative to the background.

## Introduction

There has been accumulated psychophysical evidence since a seminal report by Melcher and Morrone (2003) that visual information across saccades is integrated in a spatiotopic manner (Melcher and Morrone, 2003; Burr and Morrone, 2012; Zimmermann et al., 2013). Such transsaccadic integration could be achieved assuming a craniotopic coordinate system. Real craniotopic neurons, whose receptive fields (RFs) were fixed to head-centered coordinates regardless of eye positions in the orbit, were found in the anterior bank of the parieto-occipital sulcus (POS; area V6A; Galletti et al., 1993) and in the ventral intraparietal (VIP) area (Duhamel et al., 1997). These craniotopic neurons are believed to utilize the activities of parietal neurons whose retinotopic responses are modulated according to eye positions relative to the head (Andersen and Mountcastle, 1983; Andersen et al., 1985; Zipser and Andersen, 1988). In a more recent study, VIP neurons were shown to represent the direction of a moving object in the world coordinate by integrating visual motion signals with self-motion signals (Sasaki et al., 2020).

Recent psychophysical studies have shown that our brain automatically encodes an object location relative to the background in a scene (Boi et al., 2011; Lin and He, 2012; Uchimura and Kitazawa, 2013; Inoue et al., 2016b; Tower-Richardi et al., 2016; Chakrabarty et al., 2017; Nishimura et al., 2019). For example, a target position for reaching movement is automatically and instantaneously encoded relative to a large rectangle in the background (Uchimura and Kitazawa, 2013; Inoue et al., 2016b; Nishimura et al., 2019). This body of literature points to the existence of nonegocentric coordinates anchored to the background of the scene, distinct from both retinotopic and the still egocentric craniotopic coordinates.

In search of the neural basis of the background coordinate, we previously demonstrated in a functional imaging study of humans that the precuneus, a major hub of the default-mode network, was involved in encoding a target location relative to a large rectangle in the background (Uchimura et al., 2015). Two points are worth noting. First, the task in the imaging study was target discrimination (differentiating a circle from an apple), meaning that the target location was completely irrelevant to the task. Second, the involvement of the precuneus ceased when the large rectangle was replaced with a salient but smaller rectangle.

**Figure 1.**
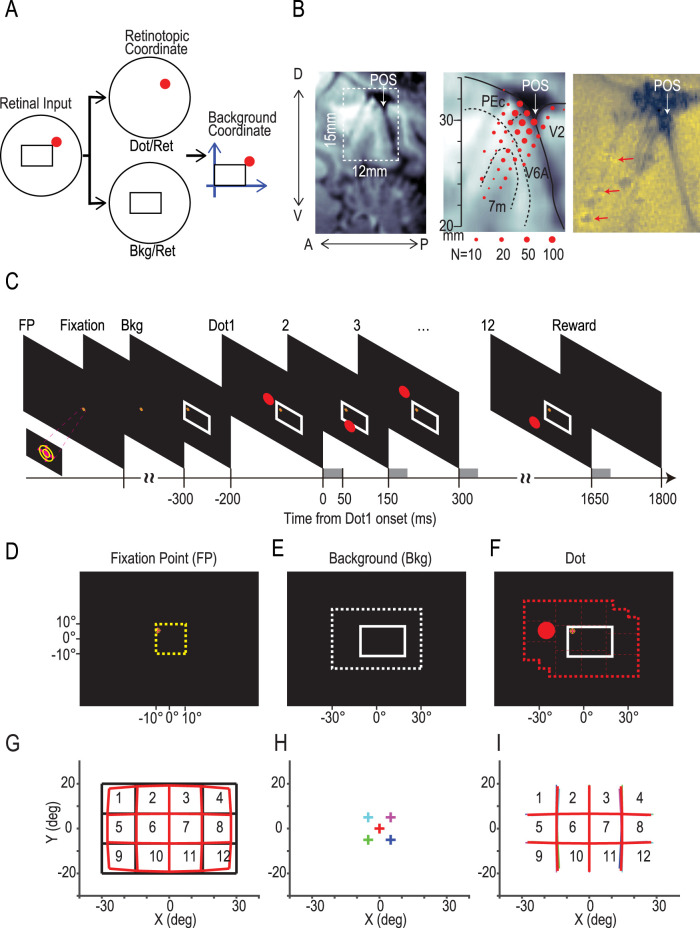
Design of the study and experiments. ***A***, A hypothesis of direct background-centric coding from retinotopic representations. A dot can be represented relative to the background (background coordinate) by relying on retinotopic representations of the dot (dot/ret) and the background (bkg/ret) without resorting to any intermediates. In this study, we examined whether and how the precuneus is involved in these processes. ***B***, Approximate locations of 942 isolated neurons around the POS are shown by dots on a typical sagittal slice of an MRI image of Monkey 1, taken 3 mm from the midline. Recordings were made in four hemispheres of three monkeys along parallel tracks that were spaced by 1 mm apart. Note that the tracks are compiled across different sagittal planes and different hemispheres of the three monkeys to show the rough locations of recordings. See Figure 2 for detailed locations. The size of each dot represents the number of isolated neurons. The anatomical subdivision (Saleem and Logothetis, 2012) of this particular slice is shown in the middle panel. PEc, area PE caudal; 7m, area 7; medial subdivision (also known as area PGm); V2, second visual area; V6A, visual area 6A. A scale on the middle panel shows the dorsoventral coordinate from the ear canal. In the right panel, arrows show MRI-detectable elgiloy deposit markings on the most anterior track. ***C–F***, A sequence of events in one trial. An FP was presented at a random location within a square zone (***D***, 20 × 20°). If the monkey fixated on the marker within 100 ms, a rectangular frame (30 × 20°, background, bkg) was presented at a random location within a rectangular zone (***E***, 60 × 40°). Then, after an interval of 200 ms, a red dot was presented sequentially at 12 different locations with stimulus onset asynchronies of 150 ms. Red dots were presented with an even probability within a zone (***F***, thick red broken line) that was defined as a union of three rectangular zones of 60 × 40° regions around the frame, center of the monitor, and the FP. Thin red broken lines show 12 equiareal subregions of the zone. Note that the edge of the monitor was invisible to the monkeys in the dark environment throughout the task period. ***G–I***, Distortions of imaginary retinotopic zones in retinal coordinate. ***G***, Imaginary 12 regions for calculating dot/ret information. These regions are rectangular on the display (black) but distorted on the retina (red). The distortion was simulated assuming that the monkey fixated on the center of the display (0, 0). ***H***, Typical fixation locations, including the center (red), and four median positions in each quadrant of the fixation target zone: (5, 5), (−5, 5), (−5, −5), and (5, −5). ***I***, Borders of retinotopic regions under typical conditions. The colors of the borders correspond to the fixation positions shown in ***H***.

In this study, we searched for background-centric visual neurons in the monkey precuneus, whose RFs were fixed to the background. We hypothesized the simplest process for calculating a stimulus position relative to the background (Fig. 1*A*): retinal images are separated into the dot and the background in the retinal coordinate (dot/ret and bkg/ret) and then combined to represent the dot location relative to the background (dot/bkg). Ultimately, we found background-centric neurons in the precuneus whose RFs were fixed to the rectangle. Furthermore, we found that the two types of retinal information (dot/ret and bkg/ret) were represented in a time-division multiplexing manner.

## Materials and Methods

### Animals

We used three (two males and one female) monkeys (*Macaca fuscata*) weighing 8.0 kg (Monkey 1, male), 5.5 kg (Monkey 2, female), and 9.0 kg (Monkey 3, male). The animals were cared for in accordance with the guidelines for the proper conduct of animal experiments established by the Science Council of Japan. All experiments were approved by the Ethics Review Committee for Animal Experimentation of the Graduate School of Frontier Biosciences, Osaka University.

### Surgery

Before training, we performed surgery under aseptic conditions to fix a head-restraining device to the skull. The monkeys were administered ketamine (10 mg/kg body weight) and an analgesic (butorphanol tartrate 0.2 mg) intramuscularly. The monkeys then received pentobarbital sodium intravenously (20 mg/kg body weight). After partially exposing the skull, the head-restraining devices were fixed to the skull with dental acrylic resin and titanium screws (2.6 mm in diameter and 5.5 mm in length). After surgery, the monkeys were administered diclofenac sodium suppositories, each containing 12.5 mg of the medication. The monkeys received antibiotics (cefazolin 50 mg/kg) intramuscularly for 1 week and given water and food *ad libitum* for >1 week after surgery. After monkeys became able to perform tasks, craniotomy was performed, and a cylindrical recording chamber was attached over the POS (Fig. 1*B*) under the same aseptic conditions as in the first surgery. The position and angle of the chamber were determined based on preoperative anatomic MRI (3 T, Prisma Fit, Siemens Healthineers) so that the axis of the cylinder (inner diameter, 18 mm) belonged to the sagittal plane 3 mm lateral from the midline and became perpendicular to the surface of the dura mater (28–35° from the vertical).

### Apparatus and task procedure

The monkeys were seated with their heads restrained in a primate chair. A visual stimulus was presented on a 75 in laser monitor (L75T, Mitsubishi) positioned 42 cm in front of the monkeys' eyes. The monitor subtended a visual angle of ∼123° (width) × 94° (height), the edge of which was invisible to the monkeys in the dark environment throughout the task period. Visual stimuli were presented at a frame rate of 60 Hz. Each trial was initiated by presenting a fixation point (FP; Fig. 1*C*, 2° magenta cross superimposed on two yellow circles with radii of 0.8 and 0.4°) at a randomly selected grid point within a square region (20 × 20°; Fig. 1*D*), where the grid was defined by 21 × 21 points with 1 mm spacing between each point. One hundred milliseconds after the monkeys' gaze got within 3° both vertically and horizontally from the FP (Fig. 1*C*, fixation), a white rectangular frame (30 × 20°; Fig. 1*C*, Bkg) was presented at a randomly selected grid point (1 mm, 0.136° spacing) within a rectangular zone of 60 × 40° (Fig. 1*E*, dotted rectangle). Two hundred milliseconds later, a red dot (5° in radius) was presented at a randomly selected grid point (1 mm spacing) within a zone (Fig. 1*F*, red broken line) for 50 ms (Fig. 1*C*, Dot 1). The dot zone was determined to cover three 60 × 40° rectangular zones around the FP, the center of the monitor, and the center of the frame in the background (Fig. 1*F*). The dot zone was further divided into 12 subregions, each of which occupied 1/12 of the dot zone, and 12 dots were presented sequentially in 1 of the 12 subregions in an order of random permutation, with 11 blank periods of 100 ms in between (Fig. 1*C*, Dots 1, 2, …, 12). We note that each dot location was determined randomly from the grid points (1 mm spacing) within each subregion. Monkeys were rewarded with juice or water if they could hold their gaze through the trial (Fig. 1*C*, −300 to 1,800 ms). We ran the same procedures in another condition (no-background condition), in which we made the background rectangle invisible by drawing its contour in black (Fig. 8*A*). The monkeys' horizontal and vertical eye positions were sampled from their right eye at 250 Hz using an infrared eye tracker (EyeLink 1000, SR Research). The timing of presentation of each visual stimulus was monitored at 1,000 Hz using a small photosensor that was fixed on the surface of the monitor. Behavioral tasks and data acquisition were controlled with TEMPO (Reflective Computing).

### Recording procedure

**Figure 2.**
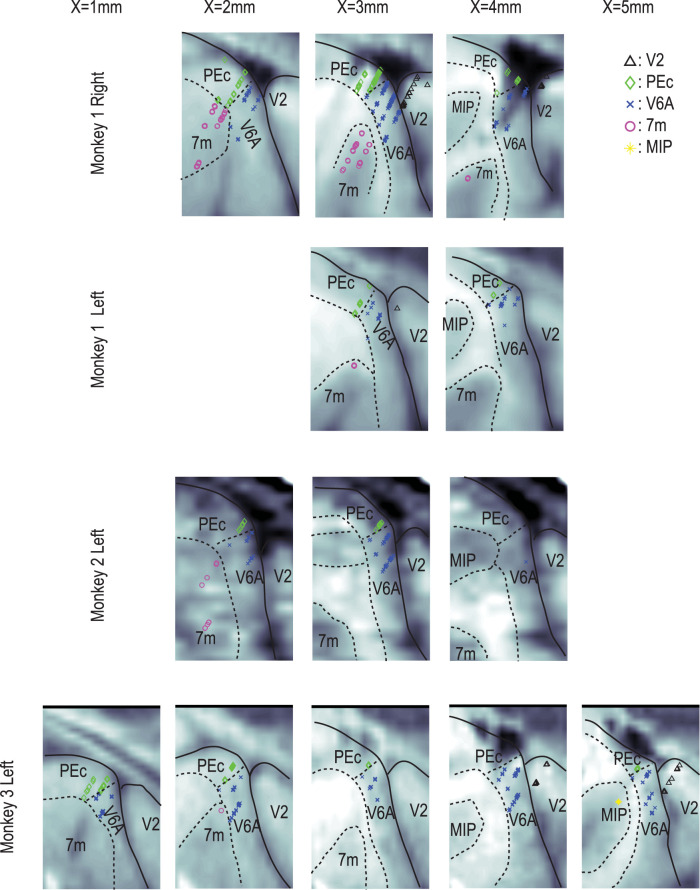
Slice-by-slice details of recording sites in each hemisphere of the three monkeys. On each sagittal MRI image, we drew contours of the gray matter and the white matter and compared them with those of an MRI and histology atlas of the macaque brain (Saleem and Logothetis, 2012) to classify recording sites into one of the following regions, V2 (second visual area), PEc (area PE caudal), V6A (visual area 6A), 7m (area 7, medial subdivision), and MIP. Each of the 942 neurons is located in one of the slices. Different colors and symbols show different regions.

Extracellular activities were recorded using a tungsten electrode (0.5–2.0 MΩ at 1 kHz; FHC), an 8-channel probe (Unique Medical, 100 µm interchannel spacing, 60 mm total length), or a 16-channel probe (U- or S-Probe, Plexon, 75 µm spacing, 80 mm total length) from the area around the POS (Fig. 1*B*). Before recording, a guide tube was manually lowered through a hole in the grid until it penetrated the dura mater. Electrodes were then inserted through the transdural guide tube using a pulse motor micromanipulator (MO-951; Narishige) mounted on the recording chamber. Raw signals from the electrodes were amplified and digitized with analog headstages (HST/8o25) and a digitizing amplifier (DigiAmp) and then stored at 40 kHz on a hard disk with a standard recording system (Omniplex, Plexon). A total of 358 penetrations (136 with a single-channel electrode and 232 with a multichannel probe) were made from four hemispheres of the three monkeys (Figs. 1*B*, 2).

### Localization of recording sites

We used a grid system with an interval of 1 mm to hold the guide tube. Thus, tracks of penetrations were parallel to each other with a minimal interval of 1 mm. To localize these tracks in the brain, we marked the most anterior track in the left hemisphere of Monkey 1 using MRI-detectable elgiloy deposit markings (Koyano et al., 2011). We marked three positions on the track (Fig. 1*B*, right panel, red arrows) by using anodic currents of 2–5 µA for 5 min passed through an elgiloy microelectrode. The positions of the track and the marks were within 1 mm of those we expected from the MRI images of the brain and the chamber and the depth of recording sites measured from the touch on the gray matter. Individual tracks of recordings were thus localized on the sagittal sections of MRI images, which were prepared every 1 mm from the midline at 1, 2, 3, 4, and 5 mm (Fig. 2). Note that the tracks in Figure 1*B* are compiled across different sagittal planes and different hemispheres of three monkeys to show the rough location of recordings. Recording sites were estimated for each hemisphere on the sagittal MRI section, as extensively shown in Figure 2. On each sagittal MRI image, we drew contours of the gray matter and the white matter (Fig. 2, dotted lines) and compared them with those of an MRI and histology atlas of the macaque brain (Saleem and Logothetis, 2012) to classify recording sites into one of the following regions: V2, PEc, V6A, 7m, and MIP (middle intraparietal area). Due to the ambiguity of ∼1 mm, the classification is not necessarily correct especially around the borders of each region. However, the assignment of identified units to V2 is most reliable because, as we advanced the electrode along the track, we passed through a silent zone of the POS before encountering neuronal units in the gray matter of V6A.

### Spike sorting

Spike activities were isolated on-line using a line discriminator implemented in Omniplex when a tungsten electrode was used. Spike activities recorded with multichannel probes were isolated off-line from the recorded signals after bandpass filtering (1,650–9,000 Hz) using an in-house spike sorting program. Briefly, the in-house program was developed in MATLAB (MathWorks) and decomposed multichannel signals into five (8-channel data) or nine (16-channel data) independent components using the FastICA (Hyvarinen and Oja, 1997). Each independent component was thresholded at three times the standard deviation (SD; −3 and +3) to choose candidates of spike waveforms (24 points, 600 µs in duration, 200 µs before and 400 s after the threshold point). These candidate waveforms were then separated into 2–9 clusters by fitting a Gaussian mixture model to the data. The number of clusters was determined using the minimum Bayesian information criterion. Finally, the experimenter judged by eye whether to accept each cluster of waveforms as isolated spike activities. Spikes that were stably recorded for >50 trials were used for later analyses.

### Quantification and statistical analysis

#### Statistical analyses of neuronal activities

**Figure 3.**
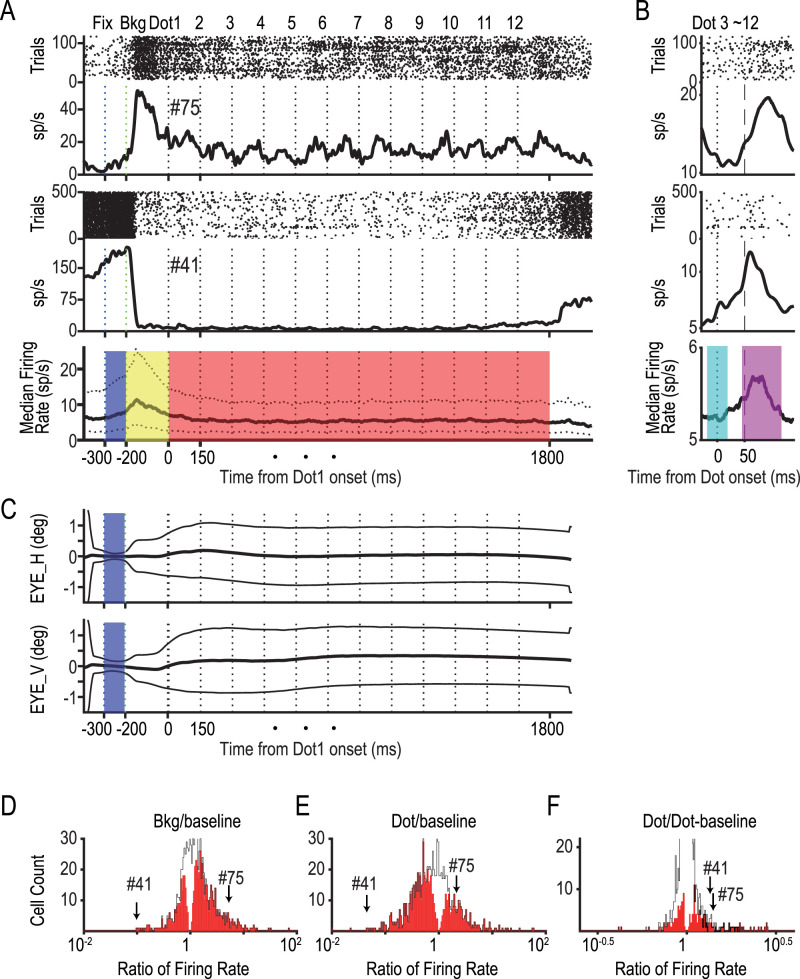
Neural activities during the task sequence. ***A***, Temporal profiles of the mean neural discharge rate of two contrasting precuneus neurons, one showing an increase (top, #75) and the other a sharp decrease (middle, #41) after the presentation of the background (bkg). Data are aligned at the time of the Dot 1 onset. Raster plots are shown at the top of each panel. The median firing rate across all 942 neurons is shown at the bottom. Dotted lines show the 25th and 75th percentiles. ***B***, Responses of two typical neurons (top and middle) and the median firing rate (bottom) aligned to the onset of dot presentations. Note the general increases in response to the dot presentations (shaded in magenta) from the dot-baseline period (cyan). ***C***, Eye-gaze locations relative to the FP. The mean (thick line) and the standard deviations (thin lines) are shown for the horizontal (Eye_H) and vertical (Eye_V) directions. Note the small standard deviations (<0.21°) during the 100 ms period from fixation (Fix, −300 ms) to the onset of background presentation (Bkg, −200 ms) that served as a baseline period (shaded in blue) in ***D*** and ***E***. ***D, E***, Distribution of the normalized responses to presentations of bkg ***D*** and dots ***E***. The mean firing rate during the test periods (shown with different colors in ***A***, yellow, bkg; red, dot) was divided by the mean firing rate during the baseline period (shaded in blue in ***A*** and ***C***). Arrows show the responses of the two neurons shown in ***A***, one with increases (#75) and the other with decreases (#41). Column colors show neuron counts with (red) and without (white) significant changes (paired *t* test; *p* < 0.005; uncorrected). ***F***, Dot responses (during the period shaded with magenta in ***B***) were compared with the baseline periods during the period of dot presentation (shaded with cyan). Note that both example neurons increased their responses (arrows).

We initially analyzed whether each neuron increased or decreased its mean discharge rate in response to the presentations of the background (bkg) and dots (Dots 1–12). For this purpose, we set a 100 ms control period after the fixation (Fig. 3*A*, bottom panel, −300 to −200 ms, shaded period in blue) and two test periods: one 200 ms period (−200 to 0 ms, yellow) after bkg presentation and one 1.5 s period from the onset of Dot 3 until after the presentation of Dot 12 (300–1,800 ms, red). We further analyzed the data by aligning the spike trains to the dot onset (Fig. 3*B*) to determine whether there was any transient response to the dot presentation. We compared the mean discharge rate during the control and test periods using a paired *t* test with a significance level of 0.005.

We then examined whether we could retrieve information on the retinotopic location of the dot (dot/ret), retinotopic location of the background (bkg/ret), and background-centric location of the dot relative to the background (dot/bkg) from the spike counts of each neuron (Kitazawa et al., 1998; Inoue et al., 2016a; Inoue and Kitazawa, 2018).

We first divided the rectangular retinotopic dot zone around the FP (60 × 40°; Figs. 1*F*,*G*) into 12 (4 × 3) equiareal subregions (15 × 13°) and numbered each from 1 to 12 (Fig. 1*G*, black). We counted the number of dots that were presented in each subregion during the period of neural recording (*n*_i_, *i *= 1, 2, 3, …, 12), and the total number of presentations *N* is given by the following:

$$N=\overset{12}{\underset{i=1}{\sum }}\;n_{i}.\;(1)$$

We then counted the number of spikes that were evoked during a certain 50 ms period ([*t* − 50, *t*] ms) in each of the *N* presentations (*C_j_*, *j *= 1, 2, 3, …, *N*) and added spike counts across presentations in each of the 12 subregions as follows:

$$m_{i}(t)=\underset{\forall j\in \{j|r(j)=i\}}{\sum }\;C_{j},(i=1,2,3,\ldots ,12),\;(2)$$

where *r*(*j*) denotes the subregion of the dot in the *j*-th presentation. If each spike occurred independent of the dot location, the expectation of the spike count *E*(*m*_i_(*t*)) is given by the following:

$$E(m_{i}(t))=M(t)(n_{i}/N),\;(3)$$

where *M*(*t*) denotes the total spike count given by the following:

$$M(t)=\overset{12}{\underset{i=1}{\sum }}\;m_{i}(t).\;(4)$$

We then calculated the mutual information *I*(*t*), which was defined as follows:

$$I(t)=-\overset{12}{\underset{i=1}{\sum }}\;(n_{i}/N)\log_{2}\;(n_{i}/N)+\overset{12}{\underset{i=1}{\sum }}\;(m_{i}(t)/M(t))\log_{2}\;(m_{i}(t)/M(t)).\;(5)$$

**Figure 4.**
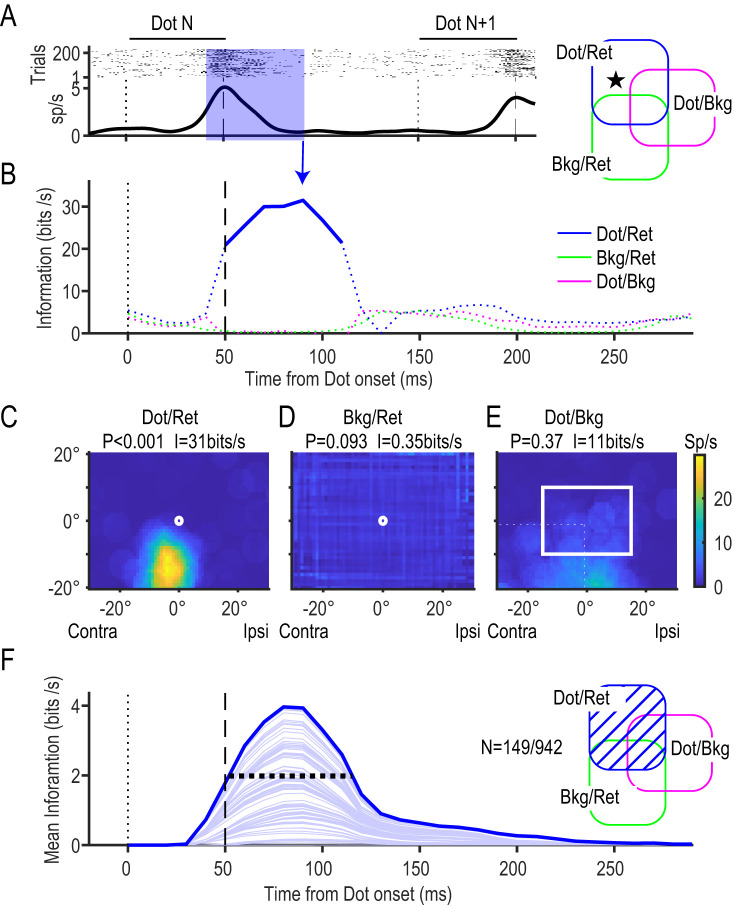
Retinotopic dot neurons (dot/ret). ***A***, Firing profiles of a pure retinotopic dot neuron (a star in the Venn diagram). Raster plots (top) and the perievent spike timing histogram (bottom) are aligned at the timing of each of the 12 dot presentations (dot N). Twelve lines of raster plots were generated for each trial. Note that the next (*N* + 1 th) dot was presented at 150 ms. ***B***, Information profiles of each dot/ret neuron. Mutual information between the retinotopic dot location and neural activity (blue line) was calculated using the retinotopic distribution of the spike counts during a 50 ms time window prior to the timing of the plot (e.g., shaded region in the top panel). A thick line shows that the information was significant (*p* < 0.005; permutation tests with *χ*^2^ statistic), and dotted lines show that the information was not significant. Information on retinotopic frame location (bkg/ret, green line) and dot location relative to the background (dot/bkg, magenta line) did not reach the level of significance. ***C–E***, RFs for the retinotopic dot location (***C***), retinotopic background location (***D***), and the dot location relative to the background (***E***) during the 50 ms period, [40, 90] ms, shown in ***A***. Note the apparent RF for the retinotopic dot location (***C***) and the lack of RFs in the others (***D, E***). *P* values (*χ*^2^ permutation test) and the information transmission rate (bits/s) are shown at the top. A color bar shows the scale of the mean firing rate. ***F***, Mean information for the retinotopic dot location. The significant information was averaged across 149 neurons with significant dot/ret information (shaded area in the Venn diagram). Intervals between thin lines show contributions from individual neurons. Note that the information started to increase 30–40 ms after dot presentation and reached a peak at 80 ms. A horizontal broken line shows the FWHM information that extended from 50 to 115 ms.

The mutual information vanishes if the spike occurs independently of the dot location. If the spikes occurred more frequently in some subregion or the neuron has some RF, the mutual information increases, up to 3.6 bits (=log_2_12 bits) at the maximum. It is worth noting that time *t* was set to the upper limit of the 50 ms time window, [*t* − 50, *t*] ms (Fig. 4*A*), and moved from 0 to 350 ms with a step size of 10 ms around the period of dot presentation (0–50 ms).

We used a *χ*^2^ statistic as follows:

$$\chi^{2}(t)=\overset{12}{\underset{i=1}{\sum }}\frac{{\left(m_{i}(t)-E(m_{i}(t))\right)}^{2}}{E(m_{i}(t))},\;(6)$$

to test whether the summed spike counts (*m_i_*(*t*)) significantly deviated from the expected counts (*E*(*m_i_*(*t*)). Dot positions were randomly permutated 1,000 times to obtain the distribution of the *χ*^2^ statistic under the null hypothesis that spikes occurred independently of the dot position. The distribution was used to obtain a *p* value of observing the actual *χ*^2^ value (or greater) under the null hypothesis. The retinotopic dot position information, *I*(*t*) of dot/ret, was judged as significant when the *p* value was <0.005 for five consecutive time steps (50 ms) and set to zero when it was not significant. We assigned a zero value to information when spike counts during a certain time window was smaller than 60, meaning that the expected number of spikes was <5 for each of the 12 subregions.

We note that the rectangular 12 subregions shown in black (Fig. 1*G*) are distorted on the retina as illustrated in red (Fig. 1*G*) when the monkey fixates on the center of the display (Fig. 1*H*, red cross). If distortions are consistent across different FPs, there is no effect on retinotopic information calculation. However, the distortions change slightly based on the fixation location. We examined four typical cases where the monkey viewed median positions in each quadrant of the fixation target zone, (+5, +5), (−5, +5), (−5, −5), and (+5, −5), as illustrated in Figure 1*H* (crosses in magenta, cyan, green, and blue). Figure 1*I* shows the borders of retinotopic regions under these conditions, with deviations from the standard being <1°, much smaller than the size of each region. We quantitatively evaluated the effect of these deviations on retinotopic information by adding Gaussian noise with an SD of 1° to the positions of the dots. The peak information and temporal profile of dot/ret information changed little: the peak decreased by <1%.

The information on the retinotopic location of the background (bkg/ret) was calculated in a similar manner, but the background zone was divided into four quadrants instead of 12 subregions because the variance of the background location was smaller than the size of the background.

Regarding the background-centric information of the dot location relative to the background (dot/bkg), dot positions were randomly permutated 1,000 times to obtain the distribution of the *χ*^2^ statistic under a null hypothesis (*H*_1_) that spikes occurred independently of the dot/bkg position. In addition, we shuffled the background locations 1,000 times to test another null hypothesis (*H*_2_) that spikes occurred depending solely on the dot/ret locations. We tested *H*_2_ because the background-centric dot location was not independent of the retinotopic dot location. For example, a dot that appeared in the left-bottom corner in the retinotopic coordinate could not be located in the right-top corner in the background-centric coordinate. Thus, any significant dot/ret information inevitably spreads into the dot/bkg information. To estimate the spread that depended solely on the dot/ret location, we calculated the dot/bkg information by shuffling the background location 1,000 times, and the mean spread of information was subtracted from the dot/bkg information. We judged that a neuron encoded significant information on dot/bkg location only when the two null hypotheses (*H*_1_ and *H*_2_) were rejected.

When we found a dot/bkg neuron, we also considered spread in the reverse direction, from the dot/bkg to dot/ret information, by shuffling the FP locations 1,000 times. When we failed to find any significant information, we increased the width from 50 to 100 ms and repeated the same procedures.

To evaluate whether the dot/ret and dog/bkg information was modulated by the location of gaze, we divided trials into four groups depending on the quadrant (the first to the fourth) of fixation on the screen. Distributions of the spike counts of the four groups were compared with the distribution of all trials by using the *χ*^2^ test.

To determine whether the RFs of dot/bkg neurons were modulated by the background location, we also divided trials into four groups depending on the quadrant of background relative to the fixation (Fig. 11*B*). Distributions of spike counts of the four groups were compared with the distribution of all trials by using the *χ*^2^ test (df = 11).

We also assessed the coding strengths of neurons for dot location in two reference frames, dot/ret and dot/bkg, using a quantitative approach suggested by Mullette-Gillman et al. (2005). Consider the evaluation of dot/ret coding strength. Initially, we computed the mean firing rates of neurons when presented with a dot in any of the 12 specific subregions on the retina, resulting in a 12-dimensional vector representing the dot/ret response for each neuron. For four distinct background locations (I–IV quadrants), we generated four corresponding response vectors (FR_1_, FR_2_, FR_3_, and FR_4_). Subsequently, we determined six correlation coefficients for the six possible pairs among these four vectors: FR_1_ with FR_2_, FR_1_ with FR_3_, FR_1_ with FR_4_, FR_2_ with FR_3_, FR_2_ with FR_4_, and FR_3_ with FR_4_. In an ideal scenario of exclusive dot/ret coding, each correlation coefficient would be one, whereas a complete lack of dot/ret coding would result in zero correlation.

The mean correlation coefficient (*r*) was calculated using the following formula:

$$r=\left(\overset{4}{\underset{\;j=2}{\sum }}\;\overset{\;j-1}{\underset{i=1}{\sum }}\frac{(FR_{i}-FR_{j})\cdot (FR_{i}-FR_{j})\;}{{\left|FR_{i}-FR_{j}\right|}^{2}}\right)/6.\;(7)$$

This calculation was performed for both dot/ret and dot/bkg coding strengths. We then computed these *r* values across a time sequence by sliding a 50 ms window from 0 to 280 ms postdot onset.

#### Evaluation of RFs

To prepare a retinotopic (or a background-centric) RF of a neuron during a 50 ms time window, we placed a dot (10° in diameter) on the retinotopic (or the background-centric) plane with a weight as large as the firing rate (spike counts/0.05 s). The background-centric plane was defined by placing its origin at the center of the square. Such dots, each representing a firing rate, were averaged across all dot presentations at each of the grid points (1° resolution) that covered the retinotopic (or the background-centric) plane. A two-dimensional Gabor function was then fitted to the RF. The function was defined as follows:

$$g\left(x,y;A,\sigma ,\gamma ,\lambda ,\psi ,c,\theta \right)=A\;\exp\;\left(-\frac{{x^{\prime }}^{2}+\gamma^{2}{y^{\prime }}^{2}}{2\sigma^{2}}\right)\cos\;\left(2\pi \frac{x^{\prime }}{\lambda }+\psi \right)+c,\;(8)$$

where

x′=(x−x0)cosθ+(y−y0)sinθ,andy′=−(x−x0)sinθ+(y−y0)cosθ.(9)

In the equations, (*x*, *y*) represents the original retinal coordinate, and (*x*′, *y*′) represents the retinal coordinate after an affine transformation with a parallel shift (*x*_0_, *y*_0_) and a rotation (*θ*). A two-dimensional Gabor function is a product of two components: one, a two-dimensional Gaussian function centered at the origin, with its axes aligned along the *x*- and *y*-axes, and a cosine wave function oriented along the *x*-axis. The center is then shifted to the actual center of the RF (*x*_0_, *y*_0_), and the axes are rotated by theta to best match the RF of the neuron. Other parameters represent the amplitude (*A*), SD of the Gaussian function (*σ*), ratio of the *x*-axis and *y*-axis of the concentration ellipse (*γ*), wavelength (*λ*), phase constant (*ψ*), and a constant (*c*). We defined the center of the RF as (*x*_0_, *y*_0_) and the size of the RF as $\sigma /\sqrt{\gamma }$. The determination coefficient (1, variance of the residual errors/variance of the RF map) was calculated to evaluate the goodness of fit.

All quantitative and statistical analyses were carried out in MATLAB (MathWorks).

#### Code accessibility

Codes and data which were used in this study will be open upon publication.

## Results

### Some precuneus neurons show increases, but others show decreases in their firing rates in response to visual stimuli

We recorded neural activities from 942 neurons, mostly in the precuneus but some in the cuneus around the POS, across four hemispheres of three monkeys (Fig. 1*B*). When the animals fixated on a point (Fig. 1*C*, FP) for 100 ms, a large rectangle (30 × 20°) was presented at a random location, which served as the background (Fig. 1*C*, Bkg). Then, a red dot was presented sequentially at 12 different locations against the background (Fig. 1*C*). It is worth noting that the animals were not required to remember any positions of the dot or the background but just to fixate on the FP for ∼2 s until they were rewarded by a drop of juice. Most neurons showed significant changes in their activity compared with the baseline in response to the presentation of a background (68%; Fig. 3*D*, Bkg) or a dot (73%; Fig. 3*E*). Interestingly, some neurons showed an increase from the baseline (Fig. 3*A*, top panel, 3*D*,*E*, #75), but others showed a decrease (Fig. 3*A*, middle panel, 3*D*,*E*, #41). An exemplified decreasing neuron showed a dramatic decrease from 150 to 5 spikes/s after the background presentation, and the mean discharge rate remained low during the period of dot presentation (Dots 1, 2, …, 12). However, by expanding the *y*-axis, we noticed that the neuron responded to each dot presentation by doubling its activity from 5 spikes/s to above 10 spikes/s (Fig. 3*B*, middle panel). Accordingly, when we compared neural activity in response to dot presentation (40–115 ms after dot presentation; Fig. 3*B*, bottom panel, magenta) against a control period from −20 to 20 ms (cyan, dot baseline), the neuron significantly increased its activity (Fig. 3*F*, #41). It is worth emphasizing that the precuneus neurons still conveyed dot information even if their mean discharge rate dropped close to zero.

### Retinotopic dot neurons

Figure 4*A* shows a neuron representing dot positions in the retinotopic coordinate. This neuron responded with a discharge rate above 8 spikes/s when a dot was presented in a contra-bottom region relative to the FP (Fig. 4*C*, peak at [−4°, −14°]) during a 50 ms period from 40 to 90 ms (Fig. 4*A*, shaded period). By observing the high discharge rate of the neuron, we obtained some information as to where the dot was presented in the retinal coordinate. This neuron represented 1.6 bits of information during the 50 ms period (31 bits/s) when we divided the retinal field into 12 equiareal regions (maximum information, 3.6 bits). A permutation test showed that the information was significant (*p* < 0.001). In contrast, this neuron did not encode significant dot position information relative to the background (*p* = 0.37; Fig. 4*E*). By moving the 50 ms time window along the time axis with steps of 10 ms, we found that significant retinal dot position information (dot/ret) appeared at 50 ms and lasted for 60 ms until 110 ms (Fig. 4*B*, blue solid trace) with a peak at 90 ms (Fig. 4*B*, arrow). In total, 149 (of 942, 16%) neurons represented significant dot position information in the retinal coordinate (dot/ret information). The mean of the dot/ret information across the 149 neurons started to increase at 30–40 ms, reached its peak at 80 ms, and subsided thereafter (Fig. 4*F*). The full-width at half-maximum information (FWHM) was 65 ms, from 50 to 115 ms (horizontal broken line).

### Background-centric dot neurons

**Figure 5.**
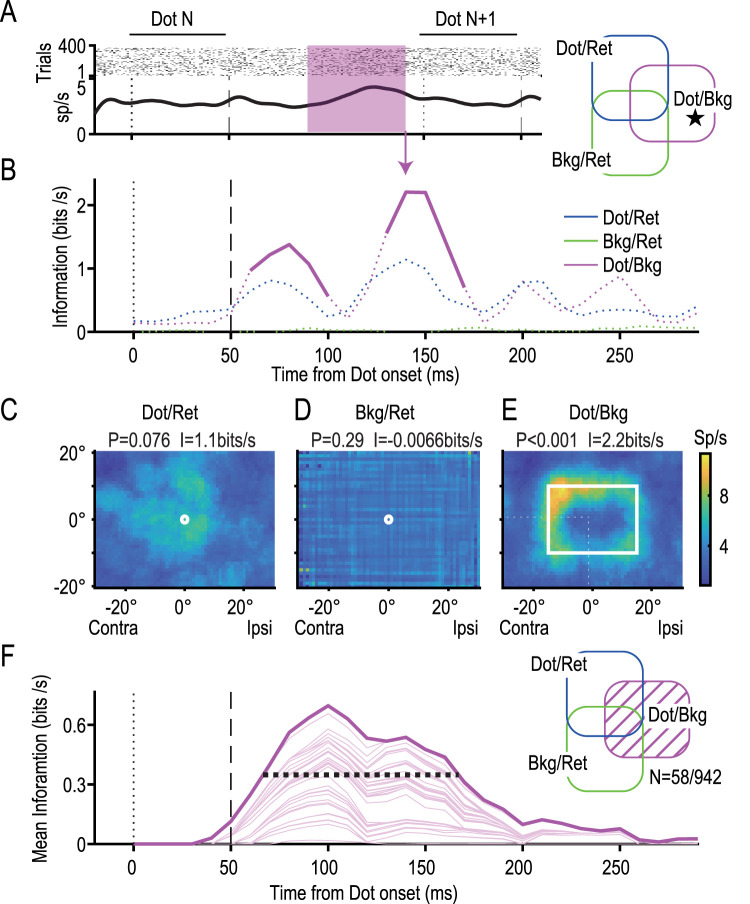
Background-centric dot neurons (dot/bkg). ***A****, **B***, Firing and information profiles of a pure dot/bkg neuron representing the background-centric dot location relative to the background. ***C***–***E***, RFs at 140 ms yielding the peak dot/bkg information (arrow in ***A***). The neuron had a background-centric RF in the left-top corner of the background (***E***) but not in retinotopic coordinates (***C***, ***D***). ***F***, Mean information on the dot/bkg location across 58 dot/bkg neurons. The FWHM extended from 65 to 165 ms.

Figure 5*A* shows a neuron that represented significant information on the dot position relative to the background (dot/bkg information). This neuron responded above 10 spikes/s when a dot was presented in the contra-top corner of the background rectangle (Fig. 5*E*) with an information transmission rate of 2.2 bits/s (*p* < 0.001). In contrast, this neuron did not encode significant information in the retinal coordinate system (Fig. 5*C*, dot/ret). The neuron showed a peak of dot/bkg information at 140 ms (Fig. 5*B*, red arrow). In total, 58 (of 942, 6.2%) neurons represented significant dot/bkg information. The mean of the dot/bkg information started to increase after 30 ms and reached its peak at 100 ms, and transmission lasted until after 200 ms (Fig. 5*F*). The FWHM was 100 ms, from 65 to 165 ms (horizontal broken line), which lagged the FWHM of the dot/ret information by 10 ms. The results show that it takes ∼10 ms to calculate the background-centric dot/bkg position after representing the dot/ret position.

### RFs of the retinotopic and background-centric dot neurons

**Figure 6.**
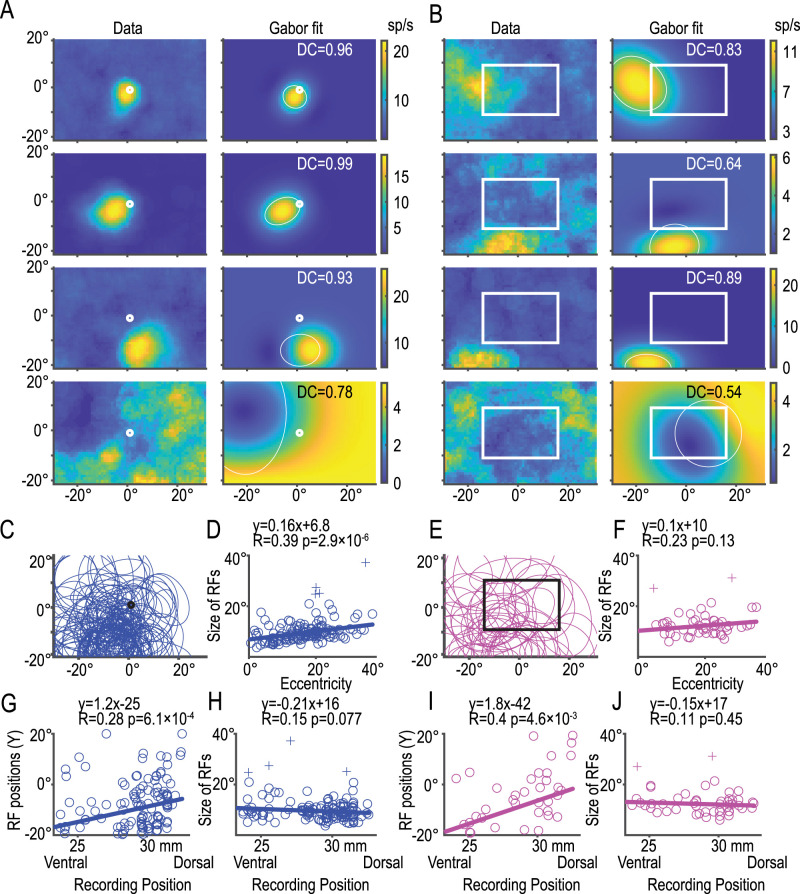
RFs of the retinotopic and background-centric dot neurons. ***A***, ***B***, Examples of retinotopic (***A***) and background-centric (***B***) RFs. RFs of four retinotopic neurons are shown in ***A*** and those of other four background-centric neurons are shown in ***B***. Two panels in each row show the actual data from a single neuron in the left and its approximation by the Gabor function in the right. White ellipses in the right panels show the size of “one-SD” of the two-dimensional Gaussian distributions of the Gabor functions. Note in ***A*** that the size of the ellipse increases as the RF moved from the center toward the periphery. ***C***, ***E***, Distributions of the retinotopic (***C***) and background-centric (***E***) RFs. ***D***, ***F***, The size of the RF plotted against eccentricity. Note that the correlation was significant in the retinotopic (***C***) but not in the background-centric (***E***) RFs. Crosses in ***D*** show outliers with residual errors >3 SD, which were excluded from regression. ***G***, ***I***, The vertical location of the RF plotted against the ventrodorsal location of neurons. Note that the correlation was significant both in the retinotopic (***G***) and background-centric (***I***) RFs: RFs moved lower as the neuron locations moved to more ventral regions. ***H***, ***J***, The size of the RF plotted against ventrodorsal location.

We then compared the spatial characteristics of the RFs in the retinal coordinate (dot/ret) with those in the background (dot/bkg). We fitted a two-dimensional Gabor function to the RF of each neuron, as shown in Figure 6, *A* and *B*. It is worth noting that a peak is often associated with a trough (Fig. 6*A*, third row) and that some neurons (∼15%) had a negative dominant RF (Fig. 6*A*, bottom row). The Gabor model captured these essential RF characteristics, as shown by the mean determination coefficients >0.5 and as large as 0.96 (dot/ret, 0.78 ± 0.17; dot/bkg, 0.72 ± 0.19 mean ± SD). We defined the RF using the concentration ellipse of the Gaussian function in the Gabor function (Fig. 6*A*,*B*, ellipses). Interestingly, the ellipses covered the bottom and contralateral quadrant (the third quadrant) in both the retinotopic (Fig. 6*C*) and background (Fig. 6*E*) coordinates. Furthermore, when we plotted the size of the RF (the geometric mean of the ellipse's axes) against its eccentricity (distance between the center of the ellipse and the origin of the coordinate), we found that the size of the RF increased linearly with eccentricity in dot/ret (Fig. 6*D*; *r* = 0.39; *p* = 2.9 × 10^−6^). In contrast, the size of the background-centric (dot/bkg) RF did not correlate with the eccentricity, which ranged from 0 to 40° (Fig. 6*F*; *r* = 0.23; *p* = 0.13). The general effect of the cortical magnification seemed to be maintained for the retinotopic RF but was somehow eliminated during the process of calculating the background-centric information.

We additionally analyzed relationships between the location and size of the RF and the stereotactic coordinates of the recording sites. We found that the location of the RF moved lower as the recording site moved in the ventral direction in both dot/ret (Fig. 6*G*) and dot/bkg (Fig. 6*I*) neurons. On the other hand, the size of the RF did not correlate with the ventrodorsal location (Fig. 6*H*,*J*).

### Some precuneus neurons initially represent dot positions in the retinotopic coordinate but later relative to the background

**Figure 7.**
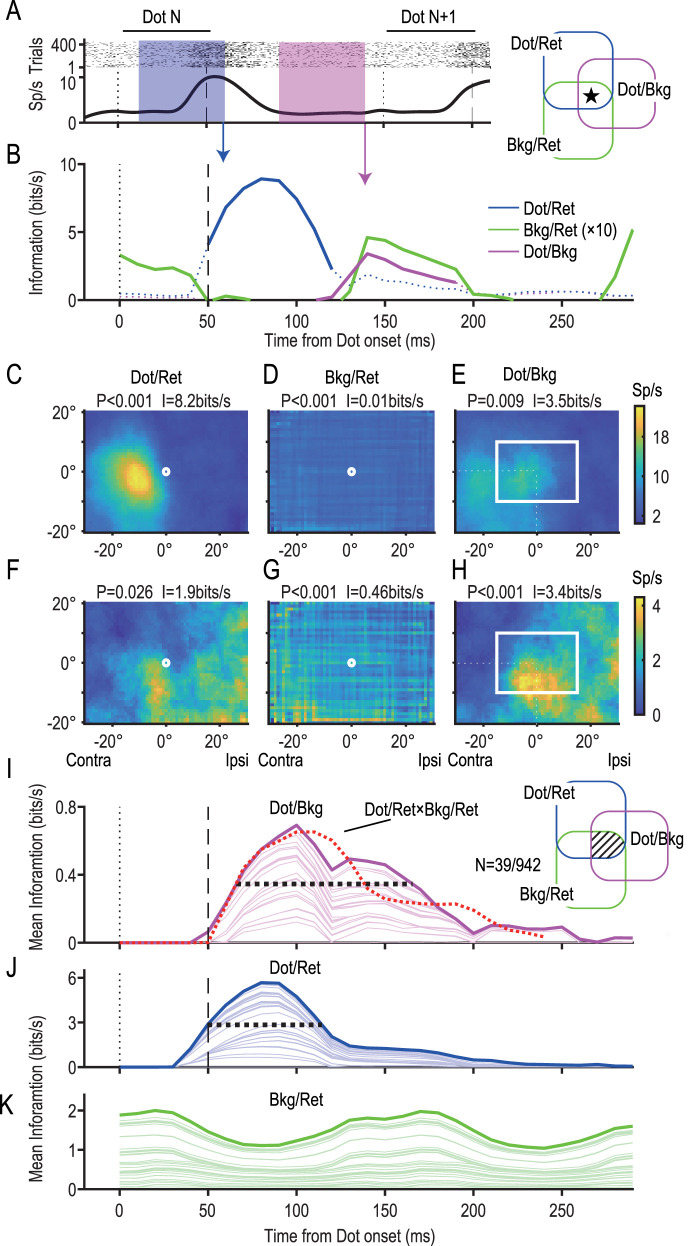
Some precuneus neurons initially represented dot positions in the retinotopic coordinate but later relative to the background. ***A***, ***B***, Firing and information profiles of a neuron with all three types of information (dot/ret, bkg/ret, and dot/bkg; a star in the triple intersection of the Venn diagram). It initially encoded bkg/ret information (green) and then dot/ret information (blue) and finally bkg/ret (green) and dot/bkg (magenta) information. The bkg/ret information is multiplied by 10 for the sake of visibility. ***C*–*H***, RFs in the initial (***C*–*E***, at 60 ms) and later periods (***F*–*H***, at 140 ms). Note that the neuron had a clear retinotopic RF at 60 ms (***C***) but later yielded a clear background-centric RF (***H***). ***I–K***, Mean information curves of 39 neurons in the triple intersection. The dotted line in red in ***I*** was obtained by multiplying two retinotopic information, dot/ret (***J***) and bkg/ret (***K***), and adding a delay of 20 ms. Note the similarity with dot/bkg information. The FWHMs extended from 65 to 165 ms for dot/bkg (***I***) and 50 to 115 ms for dot/ret (***J***).

We have shown that some neurons in the precuneus represent retinal (Fig. 4, dot/ret) information and others represent background-centric (dot/bkg) information (Fig. 5). However, these two types of information are not mutually exclusive, and some represented both (45/942, 4.8%). For example, one such neuron initially showed a clear RF in the retinotopic coordinate in particular (Fig. 7*C*, 60 ms) but later developed an RF relative to the background (Fig. 7*H*, 190 ms). The information curves (Fig. 7*B*) showed that the dot/ret information dominated from 50 to 120 ms (blue trace), but the dot/bkg information (magenta trace) took over at 120 ms and lasted until 190 ms. Furthermore, the neuron represented significant information on the background location in the retinal coordinate (bkg/ret, green trace). It is worth noting that the bkg/ret information made two peaks as if it alternated with the dot/ret information (Fig. 7*B*). These features remained when we averaged three types of information across 39 neurons that represented all three types of significant information (Fig. 7*I*–*K*). The initial dot/ret information (Fig. 7*J*) was followed by the background-centric dot/bkg information (Fig. 7*I*), and the bkg/ret information peaked before and after the dot/ret information (Fig. 7*K*).

### Two types of retinotopic information, dot/ret and bkg/ret, are represented in a multiplexing manner

In Figure 7, we observe an inverse relationship between dot/ret and bkg/ret information: as information on dot/ret emerges, there is a corresponding decline in bkg/ret information and vice versa. This phenomenon suggests two potential interpretations regarding the temporal dynamics of these complementary information streams.

The first interpretation posits that the information pertaining to the dot (figure) and that relating to the background (ground) are temporally distinct, processed in a multiplexed fashion. This notion (multiplexing of information) was proposed by previous studies (Kitazawa et al., 1998; Caruso et al., 2018; Jun et al., 2022). Essentially, this perspective suggests that the retinal information from the figure and the ground are not simultaneously processed but are instead represented separately over time. The second interpretation is more straightforward, proposing that the constant retinal input from the background (bkg/ret) is merely suppressed by the transient prominence of the dot's retinal input (dot/ret).

**Figure 8.**
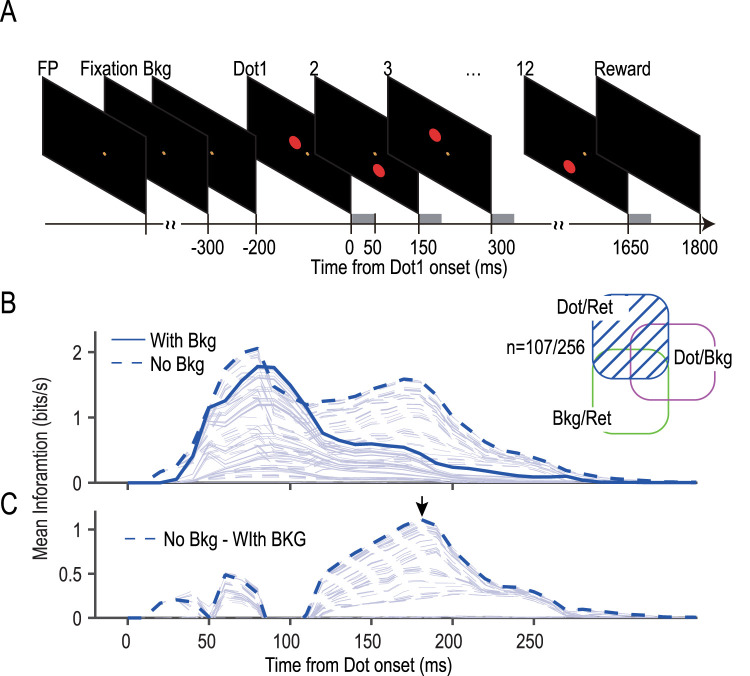
Two types of retinotopic information, dot/ret and bkg/ret, were represented in a multiplexing manner. ***A***, A sequence of events in the no-background condition. ***B***, Mean information on the retinotopic dot location in the background and no-background conditions summed across 107 dot/ret neurons. Note the increase in dot/ret information (broken line) in the absence of the background. ***C***, Temporal profile of the increase in dot/ret information in the no-background condition. Note two blocks, one up to 80 ms and another from 100 to 250 ms, with a peak at 180 ms (arrow).

To discern between these two theories, we introduced a variant in our experimental design where the background element was entirely absent (Fig. 8*A*). Under this setup, if the simpler suppression hypothesis holds, we expect the temporal dynamics of the dot/ret information to simply increase in amplitude, maintaining its original profile. Conversely, if figure and ground information are processed in a temporally multiplexed manner, dot/ret information would emerge first as figure-specific information. Subsequently, during the phase initially occupied by bkg/ret information (when background elements were present), it would reappear, this time substituting for the ground information.

We conducted recordings from 256 neurons under two different conditions: one presenting a background (Fig. 1*C*) and another without any background (Fig. 8*A*). Notably, the information profile that displayed a single peak in the presence of a background (Fig. 8*B*, solid line) evolved into a double-peak profile in the absence of the background (dotted line). The difference between the two profiles revealed two distinct phases: an initial, stable low-activity phase spanning up to 80 ms, followed by a more pronounced phase characterized by a surge in activity from 100 to 250 ms, reaching a peak at 180 ms (Fig. 8*C*, arrow).

These findings provide compelling evidence in favor of the multiplexing hypothesis. They suggest a sequential processing of information where dot/ret (figure-specific) information prevails during the initial phase up to 80 ms, followed by the dominance of bkg/ret (ground-specific) information in the 100–250 ms timeframe. This pattern is indicative of time-division multiplexing, where distinct types of retinal information are processed in separate, nonoverlapping temporal segments.

### Hybrid coding of dot/bkg and dot/ret information in dot/bkg neurons

**Figure 9.**
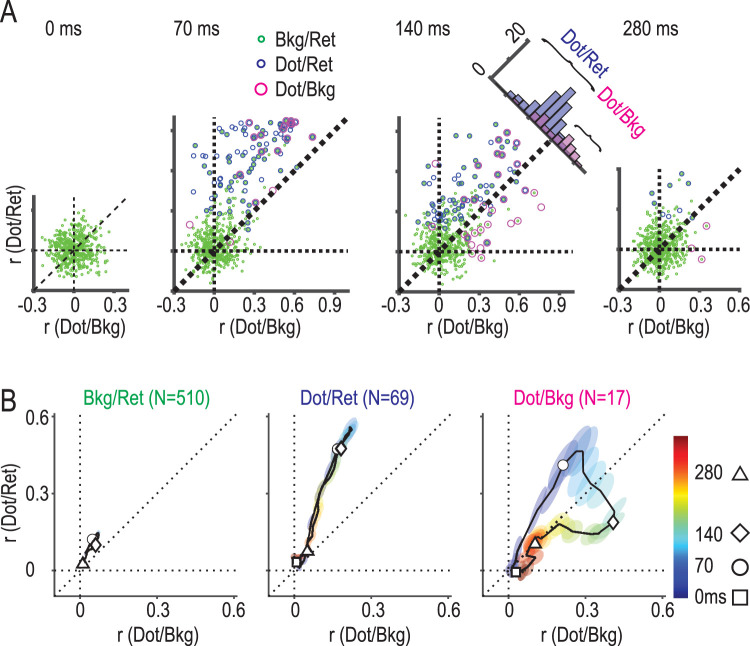
Temporal profiles of hybrid coding in dot/ret, dot/bkg, and bkg/ret neurons. ***A***, Correlation coefficients representing dot/ret (ordinate) and dot/bkg (abscissa) information in the hybrid coding analysis, plotted for individual neurons at 0, 70, 140, and 280 ms from the dot onset. Different symbols represent neurons with significant bkg/ret (green), dot/ret (blue), and dot/bkg (magenta) information, each calculated independently of the hybrid coding analyses at each time period. At 70 ms, most dot/ret neurons (blue) were distributed above the diagonal, showing the dot/ret dominance, while at 140 ms some dot/bkg neurons (magenta) crossed the diagonal into the dot/bkg dominant zone. The histogram shows the number of dot/ret and dot/bkg neurons above and below the diagonal. ***B***, The mean temporal profiles of bkg/ret (*n* = 510), dot/ret (*n* = 69), and dot/bkg (*n* = 17) neurons on the hybrid coding plane. These neuron groups were defined at 140 ms as those with significant bkg/ret information (*n* = 510), with dot/ret information and above the diagonal (*n* = 69), and with dot/bkg information and below the diagonal (*n* = 17). Different symbols show at 0 (square), 70 (circle), 140 (diamond), and 280 (triangle) ms. Colored ellipses show “standard errors of the mean” of two-dimensional Gaussian distributions estimated every 10 ms along the timeline. Note that dot/bkg neurons make a right turn at 90 ms toward the dot/bkg dominant zone, followed by a circular path deep into the zone over 120–200 ms. The circular path made a marked contrast with the linear path of the dot/ret neurons.

We have shown that some precuneus neurons initially represented dot/ret information and subsequently represented dot/bkg information in a multiplexing manner (Figs. 7, 8). To corroborate these findings from a different perspective, we employed a hybrid coding analysis (Mullette-Gillman et al., 2005, 2009; Caruso et al., 2021). We evaluated dot/ret coding and dot/bkg coding of the precuneus neurons in a temporally sequential manner by sliding a 50 ms time window along the time axis (Fig. 9). The coding strength was quantified by the mean correlation coefficient (*r*) across four different background locations on the retina, under the assumption that pure dot/ret or dot/bkg responses would be independent of background location (see Materials and Methods for detail). The coefficient takes a maximum value of one, indicating utmost stability in coding, and drops to zero when the coding is absent.

Consider, for instance, the 50 ms window set from −50 to 0 ms, just prior to the onset of dot presentation (Fig. 9*A*, 0 ms). During this interval, 485 out of 942 neurons exhibited significant bkg/ret information according to our information analysis. These 485 bkg/ret neurons clustered near the origin of the dot/ret and dot/bkg coding plane, confirming that they did not encode either dot/ret or dot/bkg information. In the subsequent time window from 20 to 70 ms, many neurons (*n* = 96) began encoding dot/ret information, depicted by blue circles. These dot/ret neurons distributed mostly along the *y*-axis (dot/ret axis) and over the triangular region above a diagonal line (*y* = *x*), indicating a predominance in dot/ret coding over dot/bkg coding. At 140 ms (the time window from 90 to 140 ms), the number of dot/bkg neurons increased from 19 at 70 ms to 30 (red circles). Many of them (*n* = 17) transitioned across the diagonal line into the dot/bkg dominant zone. Lastly, at the 280 ms time window, most neurons ceased coding for either dot/ret or dot/bkg information.

To compare the mean trajectories of bkg/ret, dot/ret, and dot/bkg neurons, we defined three groups at 140 ms (Fig. 9*A*, 140 ms): bkg/ret neurons (*n* = 510, green dots), dot/ret neurons (blue circles) in the dot/ret dominant zone above the diagonal (*n* = 69), and dot/bkg neurons (red circles) in the dot/bkg dominant zone (*n* = 17). The mean trajectory of the bkg/ret neurons, shown in the left panel of Figure 9*B*, was anchored to the origin. In contrast, the mean trajectory of the dot/ret neurons rose straight up to the peak point (0.2, 0.6) at ∼90 ms and returned along the same linear path back to the origin. The mean trajectory of the dot/bkg neurons moved linearly up to 90 ms along a slightly shallower path but then made a right angle turn to cross the diagonal border into the dot/bkg dominant zone. It reached the furthest point into the dot/bkg zone at 140 ms and returned to the diagonal at ∼200 ms. The abrupt turn of the trajectory at ∼90 ms appeared to mark a change from a dot/ret dominant period to a period dominated by dot/bkg, during which dot/ret information is combined with bkg/ret information to yield dot/bkg information. The results of these hybrid coding analyses provided further evidence supporting the multiplexing hypothesis.

### Dot/ret and dot/bkg RFs generally do not depend on the gaze position

**Figure 10.**
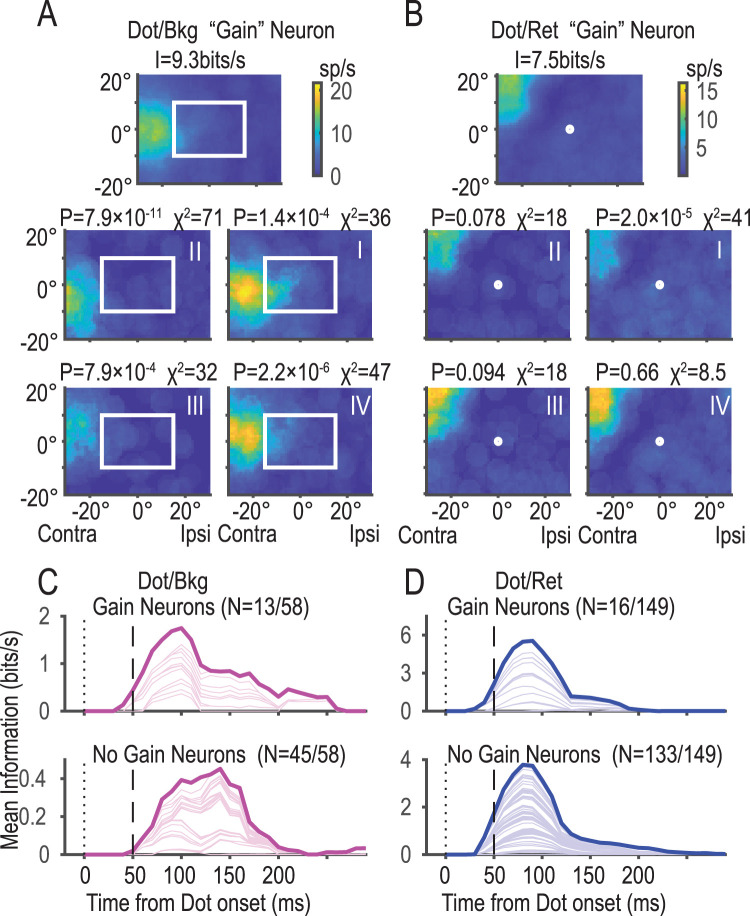
“Gain” neurons whose activities were modulated by the fixation location (eye position relative to the orbit). ***A***, An example of a dot/bkg “gain” neuron. The top panel shows the dot/bkg RF obtained using all trials. Each of the four panels below shows RF obtained using trials in which fixation fell in one of the four quadrants (I–IV). Note that activation was enhanced when the monkey viewed ipsilateral quadrants (I, IV). *P* values and *χ*^2^ statistics (df = 11) of comparison with the original are indicated above the respective panels. ***B***, An example of a dot/ret “gain” neuron. Activation was enhanced when the fixation fell in the bottom quadrants (III, IV). ***C***, ***D***, Mean information of “gain” neurons (top) and “no-gain” neurons (bottom). Note the similarity of the temporal profiles. Gain neurons were a minority in both dot/bkg neurons (22%, 13/58) and dot/ret neurons (11%, 16/149).

We further examined whether the dot/ret and dot/bkg RFs was modulated by gaze location (Fig. 10). Some dot/bkg neurons (13/58, 22%) and dot/ret neurons (16/149, 11%) showed significant changes, but the majority did not. However, the mean dot/bkg information of the neurons with modulation (gain neurons) was twice as large as that of no-gain neurons (Fig. 10).

### RFs of dot/bkg neurons are independent of background location in half of the cases

**Figure 11.**
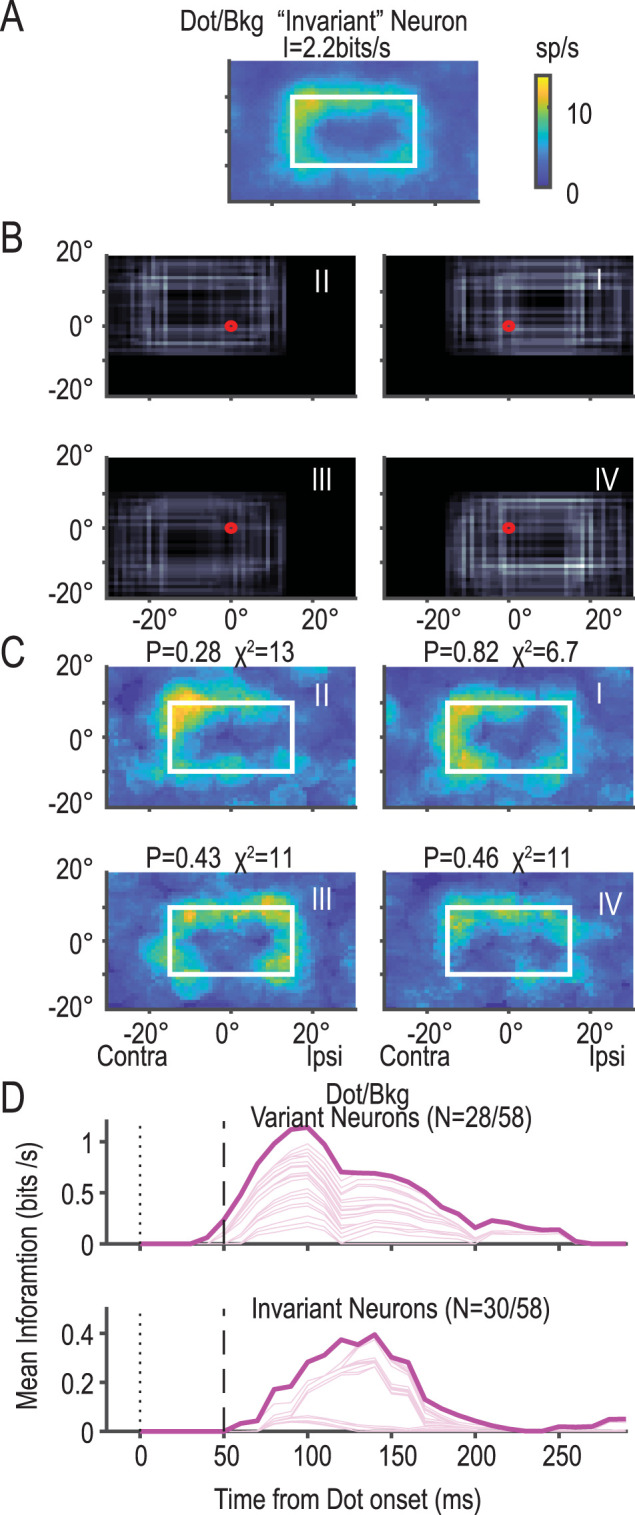
Translation invariance in dot/bkg neurons relative to background movement across the retina. ***A***, An example of a translation-invariant dot/bkg neuron, with its RF located in the top-left corner of the background. ***B***, Positions of the background are categorized into four quadrants (I–IV) in the retinal coordinate system, based on the center of the background. ***C***, The RFs of the dot/bkg neurons are depicted according to the quadrant of the background on the retina. Note the similarity of these fields to the original shown in ***A***. *P* values and *χ*^2^ statistics for each comparison are displayed above their respective panels. ***D***, The mean information for variant neurons (28 out of 58) is presented at the top, while that for invariant neurons (30 out of 58) is shown at the bottom.

We assessed whether the RFs of dot/bkg neurons were modulated by the background location, which varied widely across the four quadrants of the retina (Fig. 11). Consider, for instance, a dot/bkg neuron that produced a RF in the top-left corner of the background (as shown in Figs. 5*E*, 11*A*). When analyzing four RFs—each derived from a quarter of the data with the background in a different retinal quadrant (Fig. 11*BI*,*II*,*III*,*IV*)—we found them to be essentially comparable to the original (Fig. 11*C*; *χ*^2^ test; df = 11; *p* > 0.2). Neurons with RFs unaffected by changes in background position are termed “translation-invariant.” Nearly half of the dot/bkg neurons (52%, 30/58) exhibited this translation invariance, while the remainder did not (48%, 28/58). The mean information for translation-invariant neurons increased gradually, peaking at 120 ms, a slower rise compared with the variant neurons (Fig. 11*D*).

### Dot/bkg information is available from Dot 1 and thereafter

**Figure 12.**
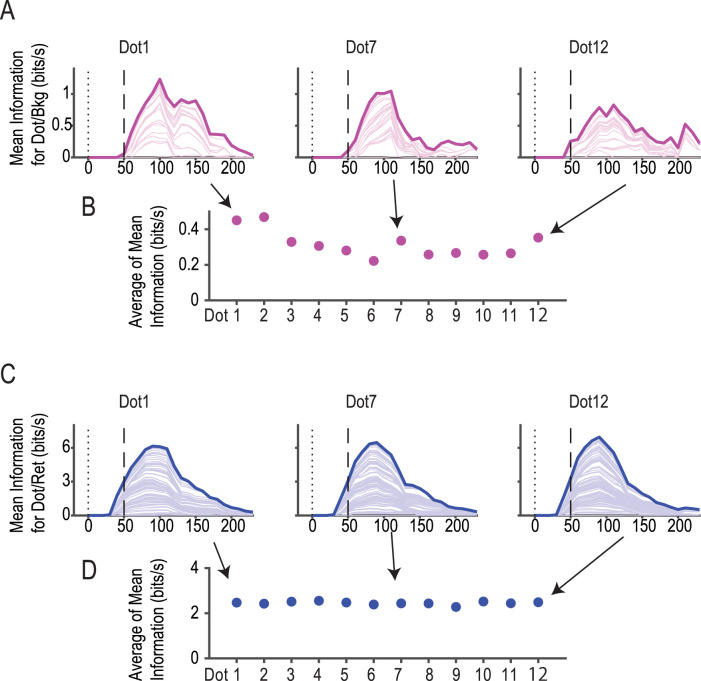
Dot/bkg (***A***, ***B***) and dot/ret (***C***, ***D***) information calculated for each of the 12 dot presentations. ***A***, ***C***, Mean dot/bkg (***A***) and dot/ret (***C***) information calculated for Dots 1, 7, and 12. ***B***, ***D***, Mean information integrated over 0–230 ms for each dot presentation. Note that dot/bkg (***B***) and dot/ret (***D***) information was almost constant across Dots 1–12.

We also examined how long it took for dot/bkg information to build up after presentation of the background. Interestingly, dot/bkg information was available from Dot 1 with a latency of 50 ms from the dot onset (Fig. 12*A*), and the information maintained a similar level thereafter (Fig. 12*B*). The results show that background-centric information was available 250 ms after the onset of presentation of the rectangle in the background.

### Relationships, localization, and firing patterns of dot/ret, bkg/ret, and dot/bkg neurons

**Figure 13.**
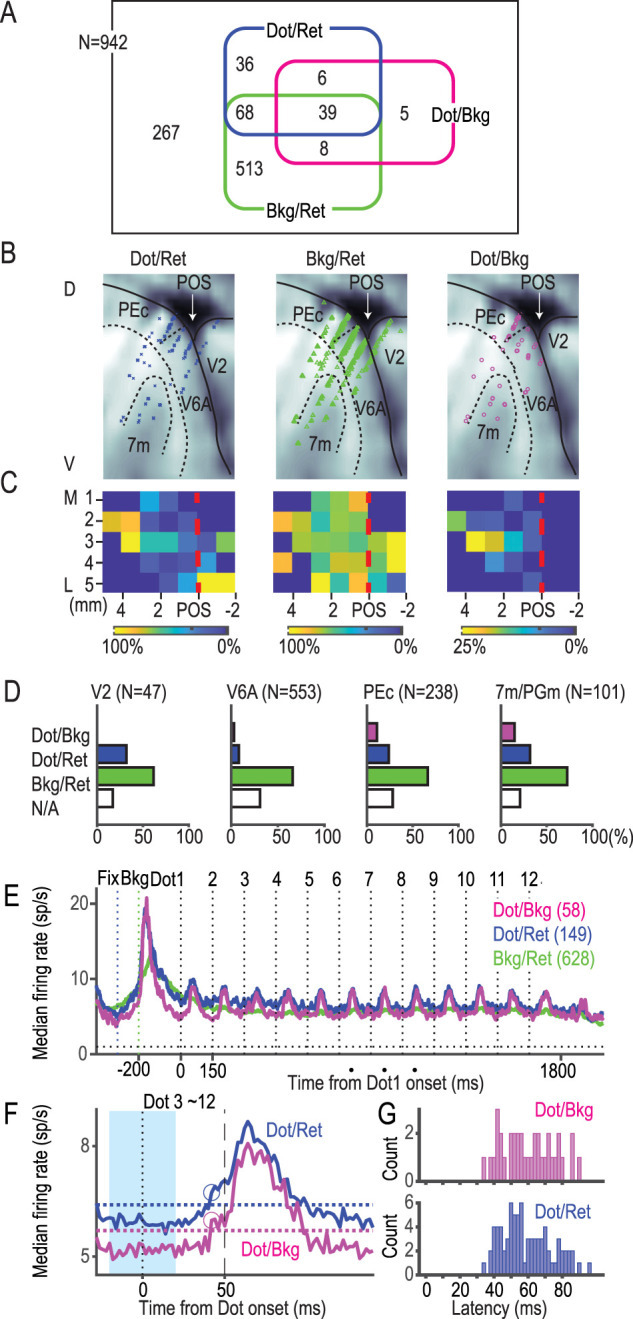
Relationships, localization, and firing patterns of dot/ret, bkg/ret, and dot/bkg neurons. ***A***, A Venn diagram showing the number of neurons in each cell group. ***B***, ***C***, Anatomical distributions of the three cell groups compiled across four hemispheres of three monkeys. Lateral (***B***) and top views (***C***). The anatomical subdivision in ***B*** applies to the slice 3 mm from the midline. See Figure 2 for slice-by-slice details. Abbreviations are the same as those in Figure 1*B*. Colors in ***C*** show the percentage of neurons classified as a particular group among neurons identified in the same grid. ***D***, The percentage of neurons with three types of information in each subdivision. Three MIP neurons, classified as bkg/ret neurons, are not shown. ***E***, Neural activities of the three groups of neurons. Conventions are the same as those in Figure 3. ***F***, Neural activities of dot neurons (dot/ret and dot/bkg). Horizontal dotted lines represent the threshold level, defined as the baseline plus four times the SD. The baseline and SD were determined from data during a control period between −20 and +20 ms (cyan shading). The onset latencies were 42 ms for both dot/ret and dot/bkg neurons, indicated by blue and magenta circles. ***G***, Distributions of onset latencies among individual dot/bkg and dot/ret neurons. Note that some neurons exhibited latencies below 40 ms.

We examined whether two types of information in the retinal coordinate (dot/ret, bkg/ret) or one in the background-centric coordinate (dot/bkg) were represented in each of the 942 neurons. A Venn diagram (Fig. 13*A*) shows that 72% (675/942) of neurons encoded at least one of the information types: 58 neurons (6.2%) encoded dot/bkg information, 149 neurons (16%) encoded dot/ret information, and 628 neurons (67%) encoded bkg/ret information. It is worth noting that 39 neurons (4.1%) encoded all three types of information. This proportion was much greater than the proportion (0.65%) under the assumption that the dot/bkg, dot/ret, and bkg/ret information were encoded independently of each other. Indeed, these neurons in the triple intersection encoded the dot/ret information and bkg/ret in a time-division multiplexing manner to yield the dot/bkg information.

Regarding the spatial distribution of each group of neurons (Fig. 13*B*–*D*), it is worth noting that all dot/bkg neurons were found anterior to the POS, that is, in the precuneus (Fig. 13*B*,*C*, rightmost panels). Accordingly, there were no dot/bkg neurons in V2 (0/47; Fig. 13*D*). Within the precuneus, the percentage of dot/bkg neurons was the largest in area 7m (15%, 15/101), followed by PEc (11%, 26/238), and V6A (3.1%, 17/553). On the other hand, neurons with retinotopic information were distributed across the POS: bkg/ret neurons were distributed widely (middle panel), but dot/ret neurons were distributed in two clusters, one around the POS and another anteriorly that overlapped with the distribution of the dot/bkg neurons.

Finally, we observed the response patterns of the three groups of neurons (Fig. 13*E*,*F*). All three types of neurons responded sharply to the presentation of the background, with a peak latency of ∼50 ms. Dot neurons (dot/ret and dot/bkg) responded to each dot presentation, with group onset latencies as short as 42 ms (Fig. 13*F*, blue and magenta circles). When we analyzed the latencies for individual neurons, 2 out of 58 dot/bkg neurons and 3 out of 149 dot/ret neurons responded within 40 ms (Fig. 13*G*). These responses conveyed three types of information.

## Discussion

In this study, we identified background-centric neurons in the precuneus of the monkey whose RFs were fixed to a large rectangle in the background. The identification itself was not unexpected because the precuneus is the top candidate region where such background-centric neurons are expected to be found (Uchimura et al., 2015). However, it is still worth emphasizing that these neurons represented background-centric information of dots (dot/bkg) with a latency as short as 40 ms by combining retinotopic information of the dot (dot/ret) and the background (bkg/ret) when the locations of the dot or the rectangle in the background was irrelevant to the ongoing task of the animals.

The automaticity and speed of background-centric neurons in the precuneus distinguish themselves from object-centered neurons in the frontal eye field (FEF) or supplementary eye field (SEF; Olson and Gettner, 1995; Olson and Tremblay, 2000; Tremblay et al., 2002; Bharmauria et al., 2020, 2021). These object-centered neurons, with RFs relative to other objects (landmarks), were identified in the FEF or SEF, while monkeys carried out some kind of memory-guided movement tasks with landmark objects in addition to a target. The latency of object-centered activity was generally much longer than 40 ms. For example, the object-centered activity of a cue location appeared with a latency of 150–200 ms after a pair of test stimuli were presented (Tremblay et al., 2002). These FEF/SEF neurons are likely useful for conducting memory-guided movement tasks, but their functionality is likely to be distinct from that of the automatic background-centric neurons in the precuneus.

Since a seminal finding in parietal neurons that retinotopic responses are modulated by the position of the eyes in the orbits (Andersen and Mountcastle, 1983; Andersen et al., 1985), such modulation is regarded as a necessary intermediate for craniotopic representation (Zipser and Andersen, 1988). Significant eye-position–dependent modulation was found in 22% of the background-centric dot/bkg neurons in this study but not in the others. We infer that precuneus background-centric neurons share craniotopic information with lateral parietal neurons but form a class of neurons independent of craniotopic neurons.

Previous studies have reported neurons in two regions whose RFs were organized independently from gaze and instead fixed to the head, one in the ventral part of V6A (Galletti et al., 1993) and the other in the VIP area (Duhamel et al., 1997). If we assume that these neurons are truly craniotopic (head-centered), the background-centric neurons in this study are distinct because the RFs of the background-centric neurons were not fixed in the craniotopic coordinate but moved with the background. However, there remains a possibility that the RFs of the “craniotopic neurons” were fixed to some large background (e.g., edges of the screen) rather than the head. To exclude this possibility, it is necessary to examine the neurons in V6A and VIP by presenting a background at different locations in the craniotopic coordinate.

It may be argued that background-centric neurons in the precuneus do not encode the dot location relative to the background but merely respond to a complex shape composed of a square and a dot, similar to neurons in the inferotemporal (IT) cortex. We can argue against the shape hypothesis from the asymmetry in the RFs for the dot in precuneus neurons: their RFs were generally located in the contralateral hemifield of the background coordinate (Fig. 6*E*). The shape hypothesis does not adequately explain this asymmetry: if one neuron prefers a dot-and-rectangle shape with a dot in the contralateral top corner of the rectangle, as shown in Figure 5*E*, we would expect to find another that prefers a dot-and-rectangle shape with a dot in the ipsilateral top corner, but such findings are significantly less probable. Assuming that the precuneus represents a background coordinate, our results show that one hemisphere dominantly represents the contralateral hemispace of the background coordinate. This representation favoring the contralateral hemispace is consistent with the general pattern of spatial representations in the parietal cortex (Gamberini et al., 2011, 2016; Duecker and Sack, 2015).

Even if these precuneus neurons respond to the combined shape of a dot and rectangle, the translation invariance across the vertical midline of the visual field, observed in half of the dot/bkg neurons (Fig. 11), distinguishes them from IT shape neurons. Although IT neurons also exhibit translation invariance, where the optimal shape remains consistent regardless of the location within their RFs (Schwartz et al., 1983; Lueschow et al., 1994; Ito et al., 1995; Logothetis et al., 1995), their RFs are generally located in the contralateral hemifield and seldom extend >5° into the ipsilateral hemifield (Op De Beeck and Vogels, 2000; Kravitz et al., 2008). By contrast, the dot/bkg neurons in our study display translation invariance across a region as large as 20° on both sides of the vertical midline, which agrees with the reported visual field representation in V6A (Gamberini et al., 2011) but distinguishes them from IT shape neurons.

We now turn to the initial schema we hypothesized (Fig. 1*A*). As we expected, precuneus neurons actually represented retinotopic information on the dot location (dot/ret) as well as that on the background (bkg/ret). What was unexpected was that the two types of retinotopic information competed with each other, perhaps for the same neural resources in retinotopic coordinates, and that the competition was solved by sequentially representing each in a time-division multiplexing manner. It is possible that the process of figure-ground separation and competition is achieved in early and higher visual cortices (V1–V4; Grossberg, 2015) and only conveyed to the precuneus. As to how the two types of retinotopic information are combined to yield background-centric information, we might need just three layers of neurons (one input, one intermediate, and one output) because a three-layer circuit was sufficient to calculate the craniotopic position of a dot from the retinotopic representation of the dot and the eye position relative to the head (Zipser and Andersen, 1988). We suggest that background-centric neurons belong to the output or intermediate layers where the two types of retinotopic information converge. We quantitatively simulated this process by multiplying the two types of retinal information (dot/ret × bkg/ret; Fig. 7*I*, dotted line in red). The simple calculation yielded a temporal profile that was remarkably similar to the temporal profile of the background-centric information (dc = 0.87), supporting the idea that the integration is achieved through a very simple process.

Finally, we discuss the use of background-centric information in the precuneus. The region in the precuneus where we found background-centric neurons involved three subregions (PEc, PGm/7m, and V6A, from anterior to posterior) that had been delineated from anatomical connectivity studies (Morecraft et al., 2004; Margulies et al., 2009). The involvement of the PEc, which has strong connections with sensorimotor regions (Morecraft et al., 2004; Margulies et al., 2016; Galletti and Fattori, 2018), suggests that background-centric information can be used for motor control in general. In addition, the V6A area has been implicated in the control of reaching/grasping movements (Galletti et al., 1997, 2005; Fattori et al., 2001, 2005, 2017; Kutz et al., 2003).

Thus, it is reasonable to speculate that background-centric information is used every time we make a simple reaching movement toward a target. Indeed, several behavioral studies have shown that humans (Uchimura and Kitazawa, 2013; Nishimura et al., 2019) and monkeys (Inoue et al., 2016b) encode a target location relative to the background even when they are asked to ignore the background and simply reach for the target. Background-centric information is likely used for controlling saccades as well (Chakrabarty et al., 2017). We speculate that such automatic encoding of a target location is useful and essential for discriminating movement errors due to erroneous motor control from those due to movement of the target (Uchimura and Kitazawa, 2013; Inoue and Kitazawa, 2018).

Another possible role of background-centric neurons could be stabilizing the visual world we perceive. It is still disputed how visual stability is achieved when retinal images jump every time we make a saccade (MacKay, 1972; Wurtz, 2008; Burr and Morrone, 2012). However, it is sensible that a background-centric coordinate will provide a stable frame of perception that is unaffected by any movements of the body or body parts. Through rich connections of the area PGm or area 7 m (Passarelli et al., 2018), with the dorsolateral prefrontal cortex, the inferior parietal lobule, and the superior temporal sulcus (Morecraft et al., 2004; Margulies et al., 2016), stable background-centric information can be shared across many cortical regions that underlie our spatial perception.

The definition of the precuneus remains under debate. For instance, Margulies et al. (2009) identified three subregions—PEc, PGm, and PO—that correspond to PEc, 7m/PGm, and V6A in our study (Fig. 1*B*). According to this definition, all background-centric neurons belong to the precuneus. However, others argue that the term “precuneus” refers more strictly to the mesial part of the brain, particularly Area 7m/PGm and Area 31 (Gamberini et al., 2020). Following this stringent definition, only 26% (15/58) of background-centric neurons are located in the precuneus (7m/PGm). However, when considering the proportion of dot/bkg neurons, 7m/PGm ranks the highest (15%, 15/101), followed by PEc (11%, 26/238), V6A (3.1%, 17/553), and V2 (0%, 0/47). The difference between 7m (15%) and V6A (3.1%) is highly significant (*p* = 4.5 × 10^−7^; *z* = 5.0; ratio test), reinforcing the conclusion that the “home ground” for background-centric neurons is located in 7m/PGm, the genuine region of the precuneus. Taken together, these results suggest that 7m/PGm plays the most critical role in transforming retinotopic to background-centric coordinates.

We have shown that stable background-centric information is represented in the precuneus, which forms a major hub of the entire cortical network (Hagmann et al., 2008; Gong et al., 2009) and is located furthest from unstable sensory input (Margulies et al., 2016). It is likely that the stable background-centric information is shared with the allocentric representations in the hippocampus (Danjo, 2020; Alexander et al., 2023) through the rich functional and anatomical connections between the precuneus and hippocampal regions (Margulies et al., 2009; Yamaguchi and Jitsuishi, 2023). Whether and how background-centric information is shared and utilized by the entire cortical network merits further investigation.
